# Easily automated radiosynthesis of [^18^F]P10A-1910 and its clinical translation to quantify phosphodiesterase 10A in human brain

**DOI:** 10.3389/fbioe.2022.983488

**Published:** 2022-09-06

**Authors:** Huiyi Wei, Junjie Wei, Shaojuan Zhang, Shiliang Dong, Guocong Li, Wenqing Ran, Chenchen Dong, Weibin Zhang, Chao Che, Wenzhao Luo, Hao Xu, Zhiyong Dong, Jinghao Wang, Lu Wang

**Affiliations:** ^1^ Center of Cyclotron and PET Radiopharmaceuticals, Department of Nuclear Medicine, The First Affiliated Hospital of Jinan University, Guangzhou, China; ^2^ Center of Bariatric Surgery, Department of Gastrointestinal Surgery, The First Affiliated Hospital of Jinan University, Guangzhou, China; ^3^ State Key Laboratory of Chemical Oncogenomics, Key Laboratory of Chemical Genomics, Peking University Shenzhen Graduate School, Shenzhen, China; ^4^ Institute of Analysis, Guangdong Academy of Sciences (China National Analytical Center), Guangzhou, China; ^5^ The Guangzhou Key Laboratory of Basic and Translational Research on Chronic Diseases, The First Affiliated Hospital of Jinan University, Guangzhou, China; ^6^ Department of Pharmacy, The First Affiliated Hospital of Jinan University, Guangzhou, China

**Keywords:** phosphodiesterase 10A, positron emission tomography, automatic radiosynthesis, translational PET/MRI, human brain

## Abstract

Our previous work showed that [^18^F]P10A-1910 was a potential radioligand for use in imaging phosphodiesterase 10A (PDE10A). Specifically, it had high brain penetration and specific binding that was demonstrated in both rodents and non-human primates. Here, we present the first automatic cGMP-level production of [^18^F]P10A-1910 and translational PET/MRI study in living human brains. Successful one-step radiolabeling of [^18^F]P10A-1910 on a GE TRACERlab FX2N synthesis module was realized via two different methods. First, formulated [^18^F]P10A-1910 was derived from heating spirocyclic iodonium ylide in a tetra-*n*-butyl ammonium methanesulfonate solution. At the end of synthesis, it was obtained in non-decay corrected radiochemical yields (n.d.c. RCYs) of 12.4 ± 1.3%, with molar activities (MAs) of 90.3 ± 12.6 μmol (*n* = 7) (*Method I*). The boronic pinacol ester combined with copper and oxygen also delivered the radioligand with 16.8 ± 1.0% n. d.c. RCYs and 77.3 ± 20.7 GBq/μmol (*n* = 7) MAs after formulation (*Method II*). The radiochemical purity, radionuclidic purity, solvent residue, sterility, endotoxin content and other parameters were all validated for human use. Consistent with the distribution of PDE10A in the brain, escalating uptake of [^18^F]P10A-1910 was observed in the order of cerebellum (reference region), substantial nigra, caudate and putamen. The non-displaceable binding potential (*BP*_ND_) was estimated by simplified reference-tissue model (SRTM); linear regressions demonstrated that *BP*_ND_ was well correlated with the most widely used semiquantitative parameter SUV. The strongest correlation was observed with SUV_(50–60 min)_ (*R* ^2^ = 0.966, *p* < 0.01). Collectively, these results indicated that a static scan protocol could be easily performed for PET imaging of PDE10A. Most importantly, that [^18^F]P10A-1910 is a promising radioligand to clinically quantify PDE10A.

## Introduction

Phosphodiesterases (PDEs) are widely present in various cells and catalyze the hydrolysis and inactivation of cyclic adenosine monophosphate (cAMP) and/or cyclic guanosine monophosphate (cGMP). Moreover, they synergize with both ATP and GTP to regulate the content of these second messengers (Charbonneau et al., 1990; Beavo, 1995; Francis et al., 2011). Therefore, PDEs play a critical role in various physiological processes involving cyclic nucleotide signaling (Bender and Beavo, 2006). In mammals, different cell types express different PDEs, which can be divided into 11 different subtypes according to their distribution and respective base sequences (Omori and Kotera, 2007). One-PDE10A-is expressed less in peripheral system, however, abundantly in striatum and substantia nigra in the central nervous system (CNS) (Loughney et al., 1999; Coskran et al., 2006; Xie et al., 2006). Medium spiny neurons in the striatum are the primary input point to the basal ganglia and they integrate both dopaminergic and glutaminergic signals with cortical reception. Ultimately, this leads to the performance of associated motor and cognitive activities (Kötter, 1994; Girault, 2012). Therefore, abnormal expression and dysfunction of PDE10A would affect the signal transmission in basal ganglia circuit. This would lead to nigrostriatal neuronal pathway-related mental and functional disorders, such as schizophrenia, Huntington’s disease (HD), Parkinson’s disease (PD) and Alzheimer’s disease (AD) (Geerts et al., 2017; Pagano et al., 2019). Thus, PDE10A is considered an attractive biomarker for the diagnosis and treatment of these diseases (Wilson et al., 2017).

**FIGURE 1:**
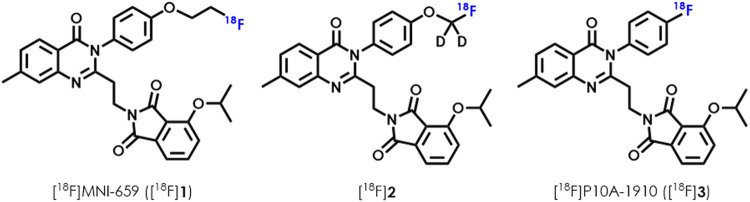
Representative PET ligands for imaging PDE10A: [^18^F]MNI-659 and their derivatives.

As a non-invasive medical evaluation method, positron emission tomography (PET) can provide valuable, *in vivo* information for exploring a host of physiological and pathological mechanisms. As a result, PET is useful in assessing the progression of related diseases, as well as help accelerate the development of targeted drugs (Gulyás et al., 1996; Cecchin et al., 2021). Given the clinical significance of PDE10A and increasing interest in PET applications, several radioligands targeted PDE10A have been reported (Laere et al., 2013; Barret et al., 2014; Russell et al., 2014; Marques et al., 2016; Mori et al., 2019; Xiao et al., 2022). In particular, a PET ligand named [^18^F]MNI659 ([^18^F]**1**, Figure 1) has been explored in healthy human volunteers and has shown high brain binding specificity in the brain as well as good pharmacokinetics. It has also been shown to have a non-displaceable binding potential (*BP*_ND_) ranging from 3.0 to 5.0 in healthy people aged 29–47 years (Barret et al., 2014). Clinical PET studies using [^18^F]**1** were further conducted on patients with HD (Russell et al., 2014; Russell et al., 2016). However, the [^18^F]fluoroethoxyl structure in [^18^F]**1** is peculiarly prone to metabolism by cytochrome-P450 monooxygenase system, resulting in unexpected radioactive aggregation in the brain and skull that interferes with imaging quantification (Russell et al., 2014). To compensate for metabolic deficiency, the side chain [^18^F]fluoroethoxy was replaced by [^18^F]fluoromethoxy-*d*_2_. The modified radioligand [^18^F]2 (Figure 1) was more stable *in vivo*, and its corresponding *BP*_ND_ was also improved (Mori et al., 2019). Nevertheless, the difficulty and complexity of radiolabeling prevented its further development (Stepanov et al., 2018; Mori et al., 2019).

Our previous work revealed the successful radiosynthesis of [^18^F]P10A-1910 ([^18^F]**3**, Figure 1) with satisfying manual labeling radiochemical yields. Its high binding specificity, good pharmacokinetics and improved metabolic stability owing to an intrinsic aryl-^18^F bond (Joong-Hyun and Pike, 2012) indicated that it was a promising radioligand for further clinical translation (Xiao et al., 2022). Here, we report a fully automated synthesis of [^18^F]**3** in a commonly used commercial GE TRACERlab FX2N radiofluorination module based on two different types of precursors. These included *i.e.*, the spirocyclic iodonium ylide (SCIDY) 2-(2-(3-(4-(((1*R*,5*S*)-4′,6′-dioxospiro [adamantane-2,2′- (Charbonneau et al., 1990; Francis et al., 2011)dioxan]-5′-ylidene)-λ^3^-iodaneyl)phenyl)-7-methyl-4-oxo-3,4-dihydroquinazolin-2-yl)ethyl)-4-isopropoxyisoindoline-1,3-dione (**5**), and the boronic pinacol ester (BPE) 4-isopropoxy-2-(2-(7-methyl-4-oxo-3-(4-(4,4,5,5-tetramethyl-1,3,2-dioxaborolan-2-yl)phenyl)-3,4-dihydroquinazolin-2-yl)ethyl)isoindoline-1,3-dione (**6**). A comprehensive quality control of [^18^F]**3** derived from these two methods was then performed to validate it for human use. Finally, a PET imaging study on healthy volunteers demonstrated that PDE10A expression in substantial nigra, caudate and putamen was sensitively quantified by [^18^F]**3** via the static scan protocol. Taken together, this work will pave the way for routine radioligand production and widespread application, as well as facile clinical translation for further diagnosis of PDE10A-related neurological disorders.

## Results and discussion

### Chemical syntheses of precursors

**SCHEME 1:**
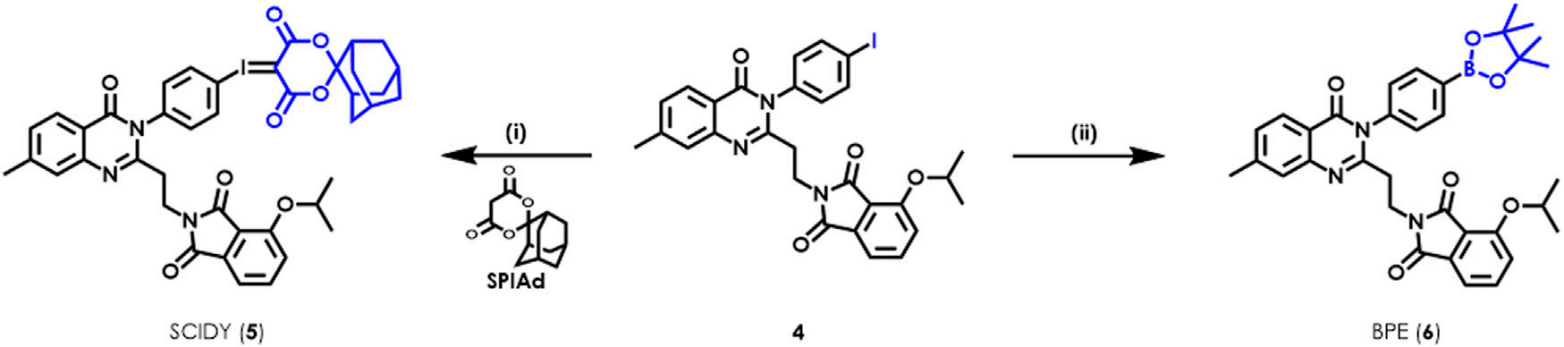
Syntheses of radiolabeling precursors SCIDY **5** and BPE **6**. Reagents and conditions: (i) oxone, TFA/CHCl_3_, room temperature, 1 h; then SPIAd, 10% Na_2_CO_3_ (aq), EtOH, room temperature, 1 h, 69% for **5**; (ii) Bis(pinacolato)diboron, Pd(dppf)_2_Cl_2_, KOAc, DMSO, 80°C, 16 h, 62% for **6**.

As shown in Scheme 1, the radiolabeling precursors SCIDY **5** and BPE **6** were prepared from the common intermediate aryl iodide **4**, which was obtained efficiently in gram scale according to our previously reported synthetic procedures (Xiao et al., 2022). Particularly, oxone was used to oxidize **4** into the corresponding iodo (III) intermediate in a solution of TFA/CHCl_3_. After evaporation of the acid solvent, the crude mixture was directly dissolved in ethanol, then coupled with SPIAd under basic conditions to deliver the desired SCIDY **5** as a colorless powder in 69% yield. Additionally, BPE **6**, a baby pink solid, was synthesized via Pd-catalyzed cross-coupling with bis(pinacolato)diboron at a comparable yield of 62%. When maintained at −78°C, both precursors remained stable radiolabeling efficiency for at least 6 months.

### Radiofluorination optimization in a GE TRACERlab FX2N module

**FIGURE 2:**
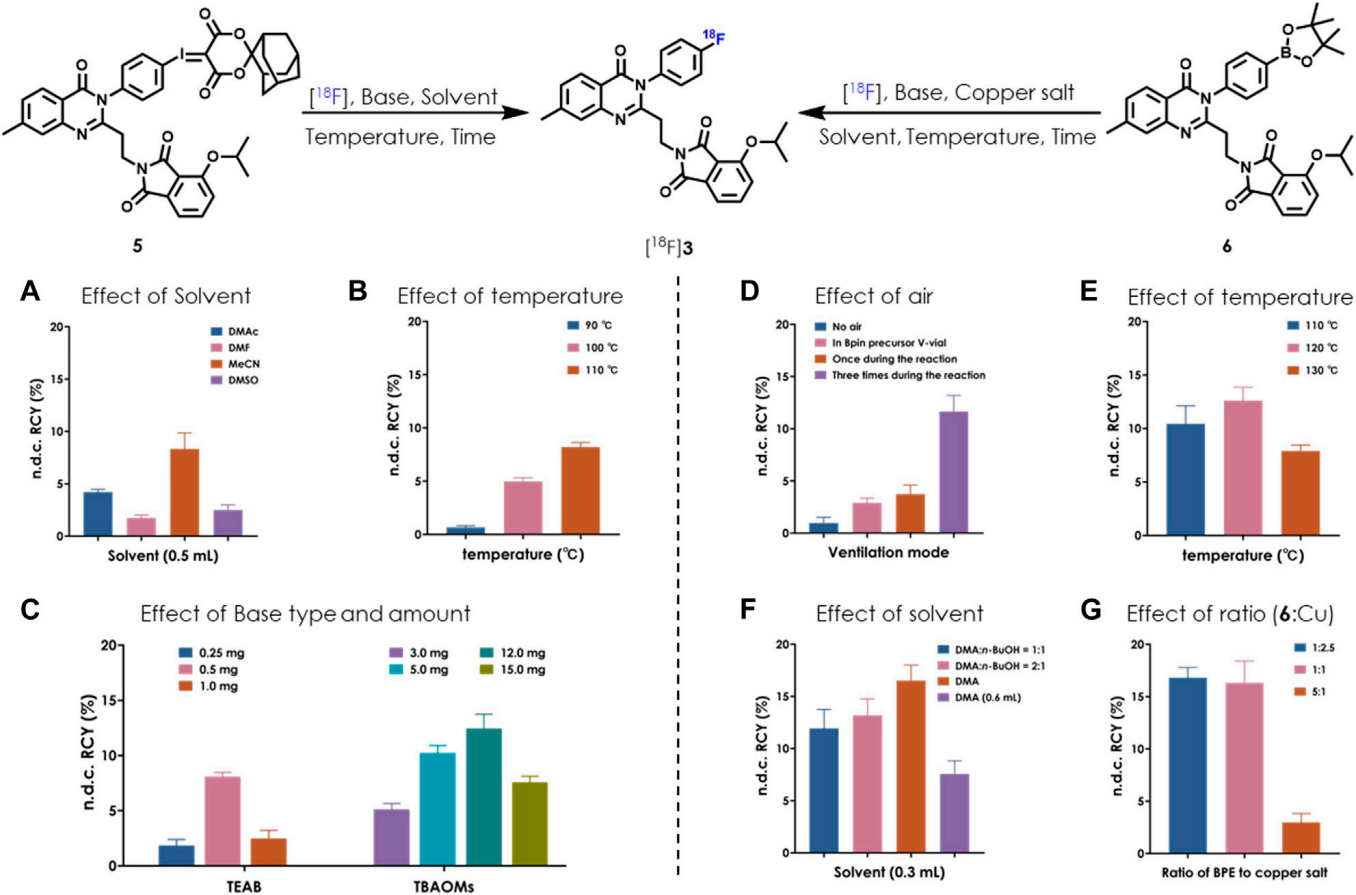
Optimization of automatic radiolabeling conditions for [^18^F]**3** based on SCIDY **5 (A–C)** and BPE **6 (D–G)** (*n* = 3). [^18^F]fluoride (14.8–18.5 GBq; 400–500 mCi) was delivered from the cyclotron to a GE TRACERlab FX2N module in each test. **(A) 5** (2 mg), TEAB (0.5 mg), 110°C, 10 min; **(B) 5** (2 mg), MeCN (0.5 ml), heated for 10 min; **(C) 5** (2 mg), MeCN (0.5 ml), 110°C, 10 min; **(D) 6** (4.25 µmol), Cu(OTf)_2_(py)_4_ (10.5 µmol), DMA:*n*-BuOH (300 μL, v/v = 2/1), TEAB (0.5 mg), 120°C, 20 min; **(E) 6** (4.25 µmol), Cu(OTf)_2_(py)_4_ (10.5 µmol), DMA:*n*-BuOH (300 μL, v/v = 2/1), TEAB (0.5 mg), heated for 20 min. Air was bubbled into the reaction three times; **(F) 6** (4.25 µmol), Cu(OTf)_2_(py)_4_ (10.5 µmol), solvent, TEAB (0.5 mg), 120°C, 20 min. Air was bubbled into the reaction three times; **(G) 6** (4.25 µmol), Cu(OTf)_2_(py)_4_ (indicated molar ratio), DMA (300 µL), TEAB (0.5 mg), 120°C, 20 min. Air was bubbled into the reaction three times.

We set out the automatic radiolabeling with SCIDY **5** according to our previously reported method (Xiao et al., 2022), resulting in only trace to low non-decay corrected radiochemical yield (n.d.c. RCY, 1∼3.2%). This was not enough to meet our demand for clinical use. This unexpected difference in outcomes between manual operation and automated performance inspired us to re-optimize the radiolabeling conditions directly on a GE TRACERlab FX2N module equipped in our facility. As shown in Figure 2A, after adjusting the type of solvent, we found that 0.5 ml MeCN gave the highest yields (8%). Reaction temperatures were then screened and 110°C was found to be stable at the tube sealing condition in the module (Figure 2B). Our continuous efforts into researching the best SCIDY strategy revealed that basic conditions may play a critical role in the balance between precursor stability and radiolabeling efficiency (Rotstein et al., 2016; Wang et al., 2017; Liang et al., 2019; Wang et al., 2020; Xiao et al., 2022). Given this, two commonly used quaternary alkylammonium salts tetraethylammonium bicarbonate (TEAB) and tetrabutylammonium methanesulfonate (TBAOMs) (Christopher et al., 2012; Inkster et al., 2016; Shi et al., 2016) were further evaluated in automation with different amounts. After, the latter was elected as the best additive at a loading of 12 mg (Figure 2C). Collectively, we identified that the combination of 12 mg TBAOMs and 2 mg SCIDY **5** in CH_3_CN at 110°C for 10 min provided the desired radioligand [^18^F]**3** with stable and high enough automatic labeling efficiencies.

Copper-induced nucleophilic ^18^F labeling, which was developed by Gouverneur’s (Tredwell et al., 2014; Taylor et al., 2017) and Scott’s group (Chu and Qing, 2014), is another strategy for radiofluorination of a non-active aromatic ring. There is easy access to the precursor BPE via Miyaura boration (Ishiyama et al., 1995), which would be complementary to SCIDY synthesis (Rong et al., 2021). Because of this, we also systemically evaluated BPE radiolabeling feasibility in our FX2N module. A big challenge in this process was controlling air bubbling; thus, we tested the effect of air and found that the highest RCYs were obtained when the mixture was ventilated three times during the radiolabeling process (Figure 2D). Heating the reaction at 120°C was also shown to be optimal (Figure 2E). By elevating the ratio of *N*, *N*-dimethylacetamide (DMA) from 50% to 100%, n.d.c. RCYs were improved to 16.5%, while increasing the solvent volume decreased RCYs dramatically (Figure 2F). Finally, the molar ratios of BPE **6** and copper salt were optimized. Although low RCYs were observed when decreasing the amount of copper salt to *ca.* 20%, comparable yields were obtained with the ratio ranging from 1:2.5 to 1:1 (Figure 2G). Together with the above results and considering efforts to minimize consumption of the precursor, we determined that marriage of 2.5 mg BPE **6**, 7 mg copper salt (molar ratio was 1:2.5), 0.5 mg TEAB and 0.3 ml DMA at 120°C for 20 min would allow for the best automatic labeling of [^18^F]**3** with the assistance of three-times air bubbling.

**FIGURE 3:**
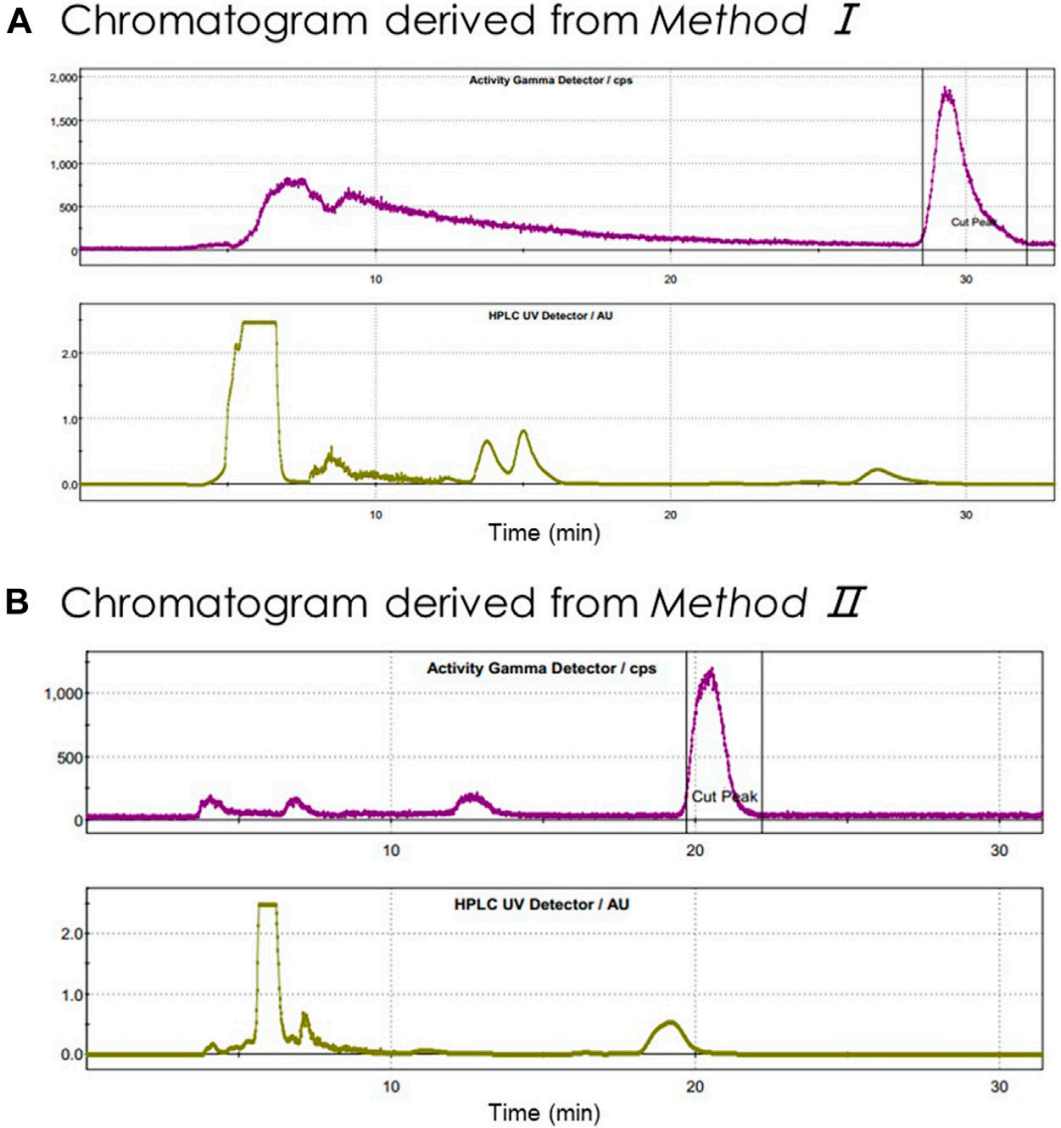
Semipreparative HPLC chromatography of crude radioligand [^18^F]**3**. Units on *y*-axis are arbitrary. Column: OSɅKɅ SODɅ, CAPCELL PAK C18, UG80 5 μm, 250 × 10 mm; **(A)** mobile phase: CH_3_CN/H_2_O = 55/45 (v/v); flow rate: 5 ml/min; Gamma (t_R_ = 29 min; purple) and UV (λ = 254 nm; yellow); **(B)** mobile phase: CH_3_CN/H_2_O = 60/40 (v/v); flow rate: 3.5 ml/min; Gamma (t_R_ = 21.8 min; purple) and UV (λ = 254 nm; yellow).

In summary, after conducting more than 100 tests in the FX2N module, we realized stable and highly efficient automatic radiolabeling of [^18^F]**3** from either SCIDY **5** (*Method I*) or BPE **6** (*Method II*). The semipreparative HPLC purification chromatography of these two methods is shown in Figure 3. The indicated radioactive peak was collected and formulated via a C18 Sep-Pak with 1.5 ml ethanol and 15.5 ml saline containing 1.55 mg ascorbic acid. The final solution was filtered by a Millex^®^-GV filter and collected in a 25 ml sterile vial. The n.d.c. RCYs obtained by these two different labeling methods were 12.4 ± 1.3% and 16.8 ± 1.0%, respectively, both of which met our requirements for further clinical application.

### Quality control and acceptance criteria for human use

According to the USP General Chapter <823> and ChP General Chapter <2321>, we conducted a comprehensive and meticulous validation and quality control for the six consecutive batches of [^18^F]**3**, three of them prepared from *Method I* and all others obtained via *Method II*. The acceptance criteria and detection results are depicted in Table 1. Immediately after formulation and sterile filtration, a series of real-time assessments were performed. The solution was observed through lead glass to ensure that it was either a colorless or light yellow and transparent liquid without any suspended particles. Using standard pH paper, pH values were determined to be between 5 and 6. The retention time of [^18^F]**3** was determined to be 5.3 ± 0.2 min as measured by an analytical HPLC system equipped with a WondaSil C18-WR column (GL Science, 4.6 × 150 mm, 5 μm), and eluted with CH_3_CN/H_2_O (7:3, v/v) with a flow rate of 1.0 ml/min. The tandem UV (*λ* = 254 nm) and Gamma detectors showed that radiochemical purities were greater than 95%, and the radioligand structure was confirmed by co-injection using standard reference (Supplementary Figures S5, S6). The above system with 20 μL of injection loop was used for cold masses estimation in the formulated solution. Molar activities were determined within a range of either 77.7–104.9 GBq/μmol (*Method I*) or 56.6–98.0 GBq/μmol (*Method II*) as calculated by a standardized curve derived from the reference compounds.

**TABLE 1:** Summary of [^18^F]**3** human use validation data.

Test	Acceptance criteria	[^18^F]3 from SCIDY (method I)	[^18^F]3 from BPE (method II)
Product yield	Report result at EOS	(1.5 ± 0.5 GBq at EOS)	(1.4 ± 0.3 GBq at EOS)
Product volume	Report result at EOS	17 ml	17 ml
Radioactive concentration	Report result at EOS	88.1 ± 29.8 MBq/ml	84.1 ± 15.4 MBq/ml
Appearance	Clear color liquid; No suspended particles	Pass	Pass
Radiochemical purity	≥95% at EOS	99%	99%
Radionuclidic identity	105–115 min	109–113 min	109–113 min
Molar activity (GBq/µmol)	Report result at EOS	88.6 ± 27.9 GBq/µmol	81.0 ± 4.3 GBq/µmol
pH	4.0–7.5	5.0–6.0	5.0–6.0
Solvents residue (USP/ICH Limits)	Acetone ≤ 0.5% v/v Ethanol ≤ 10% v/v DMA ≤ 0.041% v/v Acetonitrile ≤ 0.41 mg/ml	0.045 ± 0.016% pass — pass	0.052% pass Not detected pass
Cu	Not more than 150 μg/day	—		Not detected
Sterile Filter Integrity Test	Not less than 46 psi	>50 psi	>50 psi
Sterility	No growth observed after 14 d	Sterile	Sterile
Bacterial Endotoxin Test	Not more than 15 EU/ml	Pass	Pass

Abbreviations: EOS, end of synthesis.

By radioactivity testing every 30 min in a dose calibrator (7–8 total times), the half-life was found to range from 105–115 min. Only the 0.511 MeV and occasionally 1.022 MeV energy peaks were observed by spectrometer. No long-lived isotopes were observed by the analysis on a HPGE detector after ^18^F-decay. The formulated product was free of pyrogens (Charles River Endosafe PTS) and sterile. No copper residue was detected using ICP-MS with detection limits of 0.1 mg/L, which ensures that the tracer injection has no effect on the physiological copper level in human body (Trumbo et al., 2001; Leong et al., 2020). Volatile organic solvent analysis was conducted using GC-FID disclosing residual acetone, acetonitrile, ethanol and DMA that were well within the limits required by the International Conference on Harmonization of Technical Requirements of Pharmaceuticals for Human Use.

Although both methods could deliver qualified radiotracer, considering the radiolabeling period, synthetic cost and quality control convenience, we chose *Method I* for routine productions in clinics.

### Quantitative assessment of PDE10A in living human brains

**FIGURE 4:**
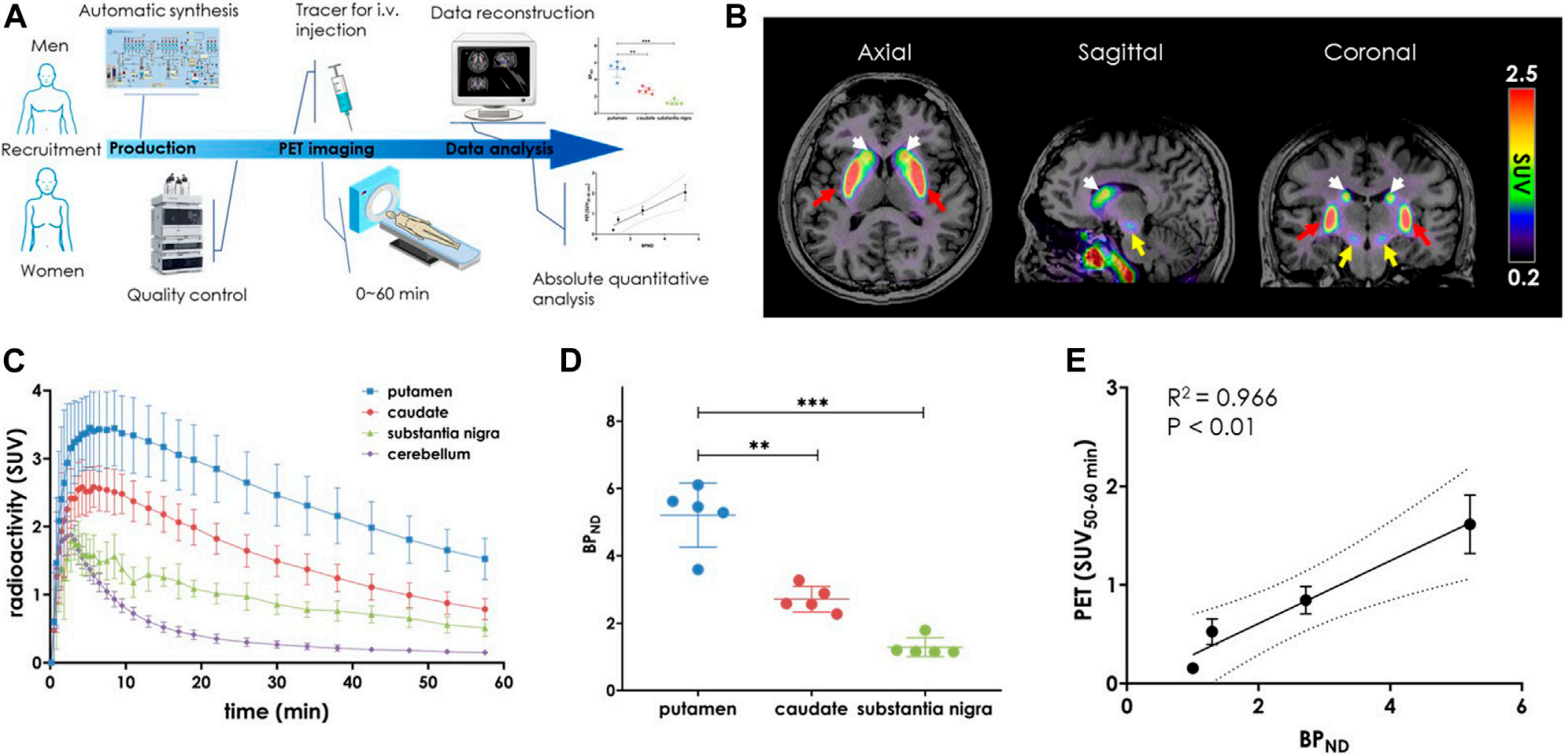
PET-MRI study of [^18^F]**3** in the living human brain. **(A)** General flow chart of the experiment including four parts: volunteer recruitment, radioligand production, PET imaging and data processing; **(B)** Averaged PET-MRI images (0–60 min) presented in the axial, sagittal and coronal views. The layers showed high uptake of the putamen (red), caudate (white) and substantia nigra (yellow). The color scale on the right side of the picture is in SUV units; **(C)** Time activity curves (TACs) of PET imaging after injection of [^18^F]**3** (*n* = 8); **(D)** *BP*_ND_ values in putamen, caudate and substantia nigra. Data are presented as mean ± SD (*n* = 8). Asterisks indicated statistical significance, ****p < 0.0001; **(E)** Correlation analysis of *BP*_ND_ with SUV_(50–60 min)_ (*R* ^2^ = 0.966, *p* < 0.01).

After signing an informed consent forms, eight healthy volunteers (HVs) aged 24–37 years (women *n* = 4, men *n* = 4) underwent brain 3.0 T magnetic resonance imaging (MRI) scan for anatomical analysis, followed by a dynamic 0–60 min PET/CT scan with injection of the radioligand [^18^F]**3**. Demographic data and radioligand information for each study are summarized in Table 2, and the outline of this translational performance is illustrated in Figure 4A. According to our efforts on automatic radiofluorination, [^18^F]**3** was produced on the day of the PET/CT scan using the GE TRACERlab FX2N module in a well-shielded hot cell. The reconstructed data were shown with averaged standardized uptake values (SUV_0–60min_) and revealed an obvious, heterogenous radioactivity distribution pattern in the human brain. The highest accumulation was found in the caudate nucleus and putamen, followed by the substantia nigra. The lowest accumulation of tracer uptake was detected in the cerebellum, implying that this region had low PDE10A expression (Figure 4B).

**TABLE 2:** Information of volunteers and administrations of [^18^F]**3**.

Number	Status	Sex	Age (years)	Weight (kg)	PET tracer administration		
					Injected dose (MBq)	Molar activity (GBq/µmol)	Cold mass (µg)
Dynamic scan (0–60 min)							
1	HV	M	33	68	260.4	43.3	3.56
2	HV	F	32	55	227.3	67.3	2.0
3	HV	M	32	63	340.7	33.7	6.0
4	HV	M	36	64	201.4	37.7	3.18
5	HV	M	37	70	286.8	19.61	8.73
6	HV	F	24	48	159.1	27.01	3.5
7	HV	F	26	51.5	196.0	18.13	6.4
8	HV	F	27	54	161.3	17.39	5.53

Abbreviations: HV, healthy volunteer; M, male; F, female.

To assess the binding specificity of [^18^F]**3** to PDE10A in human brain, regional time-activity curves (TACs, Figure 4C) were used for kinetic modeling. For this modeling, the cerebellum was used as the reference region (Liu et al., 2016; Liu et al., 2018) and non-displaceable binding potential (*BP*_ND_) was estimated by simplified reference-tissue model (SRTM). As shown in Figure 4D, the highest *BP*_ND_ were observed in the putamen, followed by the caudate nucleus (putamen vs caudate, 5.2 ± 0.9 vs. 2.7 ± 0.4, *p* < 0.001). Thus, the distribution of [^18^F]**3** in the human brain closely matched the binding patterns that had been previously observed in both rodent and nonhuman primate (NHP) brain (Xiao et al., 2022). As expected, no skull radioactivity accumulation was detected, demonstrating that [^18^F]**3** was more stable than [^18^F]**1**. No defluorination occurred *in vivo*. Promisingly and despite its small size, the nucleus substantia nigra also showed good tracer accumulation with *BP*_ND_ of 1.3 ± 0.2. This had not been previously observed in our preclinical animal neuroimaging studies (Xiao et al., 2022). To the best of our knowledge, [^18^F]**3** is the first radioligand capable of detecting PDE10A in substantia nigra in living human brain, which may pave the way for the exploration of the earliest pathogenesis in neurodegenerative diseases such as PD and HD (Chinta and Andersen, 2005; Koch and Raymond, 2019; Balestrino and Schapira, 2020).

In terms of clinical PET neuroimaging applications, either a dynamic or long time period scan would be challenging for patients due to physical or emotional causes and inescapable head movements. To verify a static scan with a reasonable duration, we then examined whether 10-min averaged SUV was practical for quantifying a target imaging signal. Comparisons between SUV_10–20min_, SUV_20–30min_, SUV_30–40min_, SUV_40–50min,_ and SUV_50–60min_ with specific binding (Supplementary Figure S8) revealed a much better relationship across the three high-uptake brain regions. Of these, the strongest relationship was observed between SUV_50–60min_ and *BP*_ND_ (Figure 4E, *R* ^2^ = 0.966, *p* < 0.01). These results indicated that the widely used SUV PET signal derived from a 10-min static scan after 50 min post-injection can be used as a routine protocol for *in vivo* measurement of PDE10A density using [^18^F]**3**.

## Materials and methods

### General considerations and study design

All commercial reagents were purchased from commercially available vendors and used as received. Unless otherwise noted, solvents were freshly dried and degassed according to the purification handbook Purification of Laboratory Chemicals before use. Analytical thin-layer chromatography (TLC) was performed on pre-coated glass-backed plates (EMD TLC Silica gel 60 F254) and visualized using either a UV lamp (254 nm) or potassium permanganate. Silica gel for flash chromatography was 300–400 mesh. NMR spectra were recorded on Bruker 400/500 MHz on Bruker spectrometers, and resonances chemical shifts (δ) were given in parts per million (ppm) relative residual solvent. Peak multiplicities are abbreviated by the following symbols: s, singlet; d, doublet; t, triplet; hept, heptahedron; m, multiplet; dd, doublet of doublets. HRMS spectra were measured on a Thermo Scientific LTQ Qrbitrap XL using ESI+.

[^18^F]Fluoride was produced by the GE Qilin MINITrace cyclotron via the ^18^O(p, n)^18^F reaction. The automatic radiolabeling was conducted in an GE TRACERlab FX2N module. Radiochemical purity and molar activity of the radioligand were determined by the HPLC (Shimadzu, Japan) with an analytical column (GL Science, WondaSil C18-WR, 4.6 × 150 mm). Radionuclide purity was tested on an energy disperse spectroscopy (Beijing Sunvic Co., Ltd.). The radioactivity of the product at the end of the synthesis was measured using a dose calibrator (Capintec CRC-55tR). The solvent and copper residue in the formulated product were tested by GC-FID (Agilent 6820) and ICP-MS (Agilent 7700x), respectively.

Clinical studies were performed in accordance with Good Clinical Practice standards, as well as in adherence with the ethical standards of the 1964 Helsinki declaration and amendments. The clinical study was approved by the Institutional Review Board (IRB) of the First Affiliated Hospital of Jinan University (Approval Letter No. KY-2022-007) (Guangdong Province Pharmaceutical Association, 2020). The healthy volunteers used in this clinical study signed informed consent forms before undergoing PET imaging with the right to know their legal guardians and welfare, as well as the nature of the study and the potential risks.

### Synthesis of SCIDY (**5**) and BPE (**6**)

*2-(2-(3-(4-(((2'S,3a'S,5'R)-4,6-dioxooctahydrospiro[* [1,3]*dioxane-2,7'-* [2,5]*methanoinden]-5-ylidene)-l3-iodaneyl)phenyl)-7-methyl-4-oxo-3,4-dihydroquinazolin-2-yl*)*ethyl)-4-isopropoxyisoindoline-1,3-dione* (SCIDY, **5**). According to our previous report (Xiao et al., 2022), SCIDY **5** as obtained was a colorless powder (69% from aryl iodide **4**). ^1^H NMR (400 MHz, DMSO-*d*_6_) δ: 7.95 (dd, *J* = 11.7, 8.5 Hz, 3H), 7.70 (t, *J* = 7.8 Hz, 1H), 7.61 (d, *J* = 8.3 Hz, 2H), 7.43 (d, *J* = 8.4 Hz, 1H), 7.33 (dd, *J* = 11.1, 7.5 Hz, 3H), 4.79 (*p*, *J* = 6.1 Hz, 1H), 3.88 (t, *J* = 7.1 Hz, 2H), 2.63 (t, *J* = 6.8 Hz, 2H), 2.43 (s, 3H), 2.35 (s, 2H), 1.94 (d, *J* = 11.9 Hz, 4H), 1.77 (s, 2H), 1.72–1.57 (m, 6H), 1.28 (s, 3H), 1.27 (s, 3H).

*4-isopropoxy-2-(2-(7-methyl-4-oxo-3-(4-(4,4,5,5-tetramethyl-1,3,2-dioxaborolan-2-yl)phenyl)-3,4-dihydroquinazolin-2-yl)ethyl)isoindoline-1,3-dione* (BPE, **6**). To the solution of compound **4** (300 mg, 1.0 eq) in 5 ml of DMSO was added KOAc (149 mg, 3.0 eq), bis(pinacolato)diboron (141 mg, 1.1 eq), Pd (dppf)_2_Cl_2_ (11 mg, 0.03 eq), and the reaction mixture was then stirred under N_2_ atmosphere at 80°C for 16 h. After cooling, the reaction mixture was diluted with EtOAc (50 ml) and washed with water (30 ml) and brine (30 ml). The organic layer was dried over Na_2_SO_4_ and concentrated to provide the crude residue which was purified by column chromatography (Hexane: EtOAc = 4:1) to deliver the desired product **6** as a baby pink solid in 62% yield. ^1^H NMR (500 MHz, CDCl_3_) δ 8.11 (d, *J* = 8.1 Hz, 1H), 7.95 (d, *J* = 8.1 Hz, 2H), 7.58–7.52 (m, 1H), 7.35–7.27 (m, 4H), 7.25–7.21 (m, 1H), 7.13 (d, *J* = 8.5 Hz, 1H), 4.67 (h, *J* = 6.0 Hz, 1H), 4.05 (t, *J* = 7.3 Hz, 2H), 2.67 (t, *J* = 7.3 Hz, 2H), 2.44 (s, 3H), 1.36 (d, *J* = 6.1 Hz, 6H), 1.33 (s, 12H) (Supplementary Figure S1); ^13^C NMR (100 MHz, CDCl_3_) δ 169.4, 168.1, 163.6, 157.0, 154.7, 148.8, 146.7, 141.1, 137.9, 137.1, 136.1, 129.7, 129.2, 128.4, 128.3, 122.3, 120.0, 116.9, 85.6, 76.5, 74.2, 36.3, 35.1, 26.4, 23.4 (Supplementary Figure S2); HRMS (m/z, ESI) calcd for C_34_H_37_BN_3_O_6_ (+) 594.2775, found 594.2768 (Supplementary Figure S3).

### Automatic radiolabeling of [^18^F]P10A-1910

A schematic diagram of the GE TRACERlab FX2N radiosynthesis module used for the production of [^18^F]P10A-1910 is shown in Supplementary Figure S4. It should be noted that there was no SPE cartridges in the positions 1, 2, and 3, where the lines were connected directly. Automated radiolabeling involved the following: 1) receiving [^18^F]fluoride in REACTOR 1 and trapping on the QMA cartridge in position 4, 2) azeotropic drying of [^18^F]fluoride in REACTOR 2, 3) radiofluorination of SCIDY **5** (*Method I*) or BPE **6** (*Method II*) in REACTOR 2, and 4) HPLC purification followed by solid-phase formulation of the final product. Radiofluorination was performed using the sequential operations indicated in the schematic diagram (Supplementary Figure S4) as follows:

Vial 6: 12 mg TBAOMs in 300 μL H_2_O and 700 μL CH_3_CN (*Method I*); 0.5 mg TEAB in 1 ml CH_3_OH (*Method II*)

Vial 7: 1 ml CH_3_CN

Vial 8: 2 mg SCIDY **5** dissolved in 1 ml CH_3_CN (*Method I*); 2.5 mg BPE **6** and 7 mg Cu(OTf)_2_ (py)_4_ dissolved in 300 µL DMA (*Method II*)

Vial 9: 1.5 ml mixed solvent (CH_3_CN: H_2_O = 3:2, v/v)

Vial 12: 10 ml H_2_O

Vial 13: 1.5 ml EtOH.

Vial 14: 15.5 ml Vc aqueous solution (100 mg/L)

Vessel 2: 80 ml sterile water

C18 1 (position 4): Sep-Pak QMA Carbonate Plus (Part No. 186004540) preconditioned with 2 ml 7.5% NaHCO_3_ aqueous solution, followed by 10 ml sterile water and 10 ml air.

C18 2 (position 5): Sep-Pak Plus Light C18 (Part No. WAT023501) activated with 5 ml EtOH, 10 ml sterile water and 10 ml air.

1) ^18^F^−^ in Vessel 1 was transferred to REACTOR 1 by vacuum pump, then passed through the QMA installed in position 4 by helium flow, where the ^18^F^−^ was trapped. The basic solution in Vial 6 was used for eluting ^18^F^−^ from QMA to REACTOR 2, where the radioactive solution was heated to 85°C for 5 min. The mixture in REACTOR 2 was again dried azeotropically by addition of 1 ml anhydrous CH_3_CN, preloaded into Vial 7, at 85°C for 3 min, then at 110°C for 5 min under helium flow and vacuum.
2) After REACTOR 2 was cooled down to room temperature, the precursor solution in Vial 8 was added. The mixture in REACTOR 2 was heated at 110°C for 10 min (*Method I*), or at 120°C for 20 min (*Method II*) during which REACTOR 2 was pumped into negative pressure and the air was passed through V25 and V37 three times.
3) The mixture was cooled to 40°C, then transferred to TUBE 2 through valves V16, VZ2, and V33. The liquid in Vial 9 was added, washing the radioactive residues from REACTOR 2 to TUBE 2 through the pipeline.
4) The crude mixture in TUBE 2 was injected into HPLC equipped with a semi-preparative column (OS∧K∧ SOD∧, CAPCELL PAK C18, UG80 5 μm, 250 × 10 mm) and eluted with CH_3_CN/H_2_O (55/45, v/v, *Method I*; 60/40, v/v, *Method II*) in Eluent 1 at a flow rate of 5 ml/min (*Method I*) or 3.5 ml/min (*Method II*) for radioligand purification. The semi-preparative HPLC chromatograms for both Methods are shown in Figure 3.
5) The indicated product portion was collected and diluted in the bottle of Vessel 2, then captured by Light C18 SPE in position 5. The cartridge was washed with 10 ml sterile water preloaded in Vial 12 to remove HPLC mobile phase and ^18^F^−^.
6) The radioligand on Light C18 cartridge was eluted with 1.5 ml EtOH in Vial 13 into collection Vessel 3, followed by 15.5 ml Vc aqueous sodium chloride solution for injection preloaded into Vial 14. The solution was transferred, via V16a, and passed through a Millipore GV vented filter (0.22 μm, SLGVR13SL) connected to a 16-gauge hypodermic needle into a sterile 25-ml dose vial (ABX) fitted with a Millex sterile filtered venting needle (0.2 μm, 25 mm, Merck).

### Quality control and analysis

An analytical HPLC (SHIMADZU, SPD-16) was performed on the system using a UV detector (*λ* = 254 nm), a gamma detector (Eckert & Ziegler, BGO Detector, B-FC-4100) and analysis software (LabSolutions Essentia). The radiochemical purity and molar activity were determined by an analytical column (GL Science, WondaSil C18-WR, 4.6 × 150 mm) with CH_3_CN: H_2_O = 7:3 (v/v) at a flow rate of 1.0 ml/min. Refer to the Supporting Information for the standard curve and calculation of molar activity (Supplementary Figure S7).

The formula for calculating injected cold mass was as follows: Injected mass dose (µg) = molecular weight (g/mol) × injected radioactivity (mCi/1000)/molar activity (Ci/µmol).

*Solvent residual testing*: Either the FFAP column (30 m, 0.25 mm, 0.5 μm) or INNOWAX column (30 m, 0.53 mm, 0.5 μm) was used with a constant hydrogen flow rate of 1.5 ml/min. Oven temperature was ramped up starting from 50°C and the front inlet was set to 240°C. A split ratio of 10:1 was used. The elution time for ethanol was approximately 1.63 min, acetonitrile was approximately 1.95 min, acetone was approximately 1.39 min, and DMA was approximately 2.23 min.

*Determination of copper residue*: Sample processing including the following: 0.5 ml was diluted to 50 ml with 2% nitric acid. Instrument conditions: RF power: 1.55 kW; Feedback power < 10 W; Sampling depth: 10 mm; Nebulizer: MicroMist; Nebulizer chamber temperature: 2°C; Argon gas flow: plasma gas 15.0 L/min, auxiliary gas 0.8 L/min, carrier gas 0.8 L/min, compensation gas 0.4 L/min; collision gas: helium, 4.3 ml/min; scanning mode: peak skipping; online internal standard element Ge, concentration 0.5 mg/L. According to these methods, the limit of quantification was determined to be 0.1 mg/L (data in house).

### PET-MRI study in living human brain

Each volunteer participating in this study signed an informed consent form. All participants underwent a complete physical exam before the study, including medical history, electrocardiogram (ECG), blood routine and chemical tests. The volunteer first underwent a brain magnetic resonance imaging examination (GE Discovery 750, Milwaukee, United States), including 3D Bravo T1 (repetition time (TR), 8.24 ms; echo time (TE), 3.24 ms; slice thickness = 1.1 mm; matrix size = 256 × 256; flip angle, 12), 3D Cor T2 Cube (TR, 3002 ms; TE, 95.328 ms; slice thickness = 1.0 mm; matrix size = 256 × 256; flip angle, 90), T2 FLAIR (TR, 5002 ms; TE, 127.316 ms; slice thickness = 1.0 mm; matrix size = 256 × 256; flip angle, 90), diffusion weighted imaging (DWI) (TR, 3000 ms; TE, 90.6 ms; slice thickness = 3.0 mm; matrix size = 128 × 128; flip angle, 90), cerebrovascular imaging (MRA) (TR, 22 ms; TE, 2.7 ms; slice thickness = 1.2 mm; matrix size = 384 × 256; flip angle, 20). Preliminary judgement from the anatomical level was conducted by two senior radiologists to make sure only healthy volunteers (HV) with normal brain function and structures were enrolled in the study.

HVs were placed in a supine position on a GE Discovery 690 PET/CT Elite scanner [^18^F]P10A-1910 (*ca.* 0.12 mCi/kg, mass dose < 10 μg) was injected into the body from the cubital vein within 20 s, followed by dynamic brain PET collection in a 3D list mode for 60 min. Reconstruction of PET raw data was 32 frames (6 × 20 s, 8 × 30 s, 4 × 1 min, 5 × 2 min, 5 × 4 min, 4 × 5 min). A specialist monitored the volunteers’ electrocardiograms (ECG) and blood pressure throughout the entire scan. After the scan, all HVs were advised to stay away from people, especially children and pregnant women, and to drink plenty of water to facilitate radioactivity excretion.

Individual MRI and PET image co-registration, and dynamic PET data processing were analyzed by PMOD (version 4.105, The First Affiliated of Jinan University). According to the biological expression of PDE10A, the caudate, putamen and substantia nigra were selected as regions of interest. Time radioactivity curves (TACs) from 0 to 60 min were generated. A simplified reference-tissue model (SRTM) was used to calculate their non-displaceable binding potential (*BP*_ND_) with the cerebellum as the reference brain region. PET signal was presented as standardized uptake value (SUV) averaged from 0–60 min (SUV_0−60_), 10–20 min (SUV_10−20_), 20–30 min (SUV_20−30_), 30–40 min (SUV_30−40_), 40–50 min (SUV_40−50_), or from 50–60 min (SUV_50−60_) post injection. SUV data were analyzed by correlating with binding potentials.

### Statistical analyses

Continuous variables were presented as mean ± standard deviation (SD). Student’s *t* test and one way analysis of variance (one-way ANOVA) tests were used for group comparisons of continuous variables. Strength and direction of associations were assessed by Spearman’s rank-order correlation. Linear regressions were performed as appropriate. A *p*-value was considered the significance level of the statistical analysis results, with *p* < 0.05 representing statistical significance. All statistical data were analyzed using GraphPad Prism (version 8.0.1).

## Conclusion

We have realized the first automatic radiolabeling of a novel PDE10A PET tracer [^18^F]**3** (designated [^18^F]P10A-1910). Using the GE TRACERlab FX2N as our operation module, either the spirocyclic iodonium ylide tailing with an adamantyl auxiliary (**5**, *Method I*) or the boronic pinacol ester (**6**) coupled with copper salt and oxygen (*Method II*) delivered the product with comparable radiolabeling yields and molar activities. Moreover, the quality control of both was fully validated for human use. A subsequent imaging study in the living human brain revealed that the radioactivity accumulated in the caudate, putamen, as well as the substantia nigra. *BP*_ND_ was strongly correlated with the PET signal indicated by SUV_50−60min_. This easy, automatic radiolabeling, good *in vivo* stability and binding potential, as well as practical protocols for use in imaging scan in clinics would facilitate widespread use of [^18^F]**3**. Critically, its use would contribute to future exploration of PDE10A-related CNS pathologies in large-scale, multicenter trials.

## Data Availability

The original contributions presented in the study are included in the article/SupplementaryMaterial, further inquiries can be directed to the corresponding authors.
