# Supplementary material for: Easily automated radiosynthesis of [18F]P10A-1910 and its clinical translation to quantify phosphodiesterase 10A in human brain
Source: Front Bioeng Biotechnol. 2022 Sep 6;10:983488. doi: 10.3389/fbioe.2022.983488 (PMC9486304; doi:10.3389/fbioe.2022.983488)
Supplement: Supplementary file 1 [file DataSheet1.docx]

**Supplementary Materials**

**Easily automated radiosynthesis of [^18^F]P10A-1910 and its clinical translation to quantify phosphodiesterase 10A in human brain**

Huiyi Wei^1†^, Junjie Wei^1†^, Shaojuan Zhang^1†^, Shiliang Dong^2^, Guocong Li^1^, Wenqing Ran^1^, Chenchen Dong^1^, Weibin Zhang^3^, Chao Che^3^, Wenzhao Luo^4^, Hao Xu^1^, Zhiyong Dong^2,5*^, Jinghao Wang^5,6*^, Lu Wang^1,5*^

^1^*Center of Cyclotron and PET Radiopharmaceuticals, Department of Nuclear Medicine, The First Affiliated Hospital of Jinan University, Guangzhou 510630, China*

^2^*Center of Bariatric Surgery, Department of Gastrointestinal Surgery, The First Affiliated Hospital of Jinan University, Guangzhou 510630, China*

^3^*State Key Laboratory of Chemical Oncogenomics, Key Laboratory of Chemical Genomics, Peking University Shenzhen Graduate School, Shenzhen 518055, China*

^4^*Institute of Analysis, Guangdong Academy of Sciences (China National Analytical Center), Guangzhou 510070, China*

^5^*The Guangzhou Key Laboratory of Basic and Translational Research on Chronic Diseases, The First Affiliated Hospital of Jinan University, Guangzhou 510630, China*

^6^*Department of Pharmacy, the First Affiliated Hospital of Jinan University, Guangzhou 510630, China*

^*^Corresponding authors.

^†^These authors contributed equally to this work.

Email Address: l_wang1009@jnu.edu.cn (L. Wang)

wangjinghao@jnu.edu.cn (J. Wang)

dongzy2008@jnu.edu.cn (Z. Dong)

**Figure S1:** ^1^H-NMR spectrum of compound BPE **6**

**Figure S2:** ^13^C-NMR spectrum of compound BPE **6**

**Figure S3:** HRMS chromatograms of compound BPE **6**

**Figure S4:** A schematic diagram of the GE TRACERlab FX2N radiosynthesis module

**Figure S5:** Chromatograms of [^18^F]P10A-1910 radiolabeled via *Method I*

**Figure S6:** Chromatograms of [^18^F]P10A-1910 radiolabeled via *Method II*

**Figure S7:** Calculation of molar activities of [^18^F]P10A-1910 at the end of formulation

**Figure S8:** PET images in healthy volunteers and linear correlation analysis between SUV and *BP*_ND_


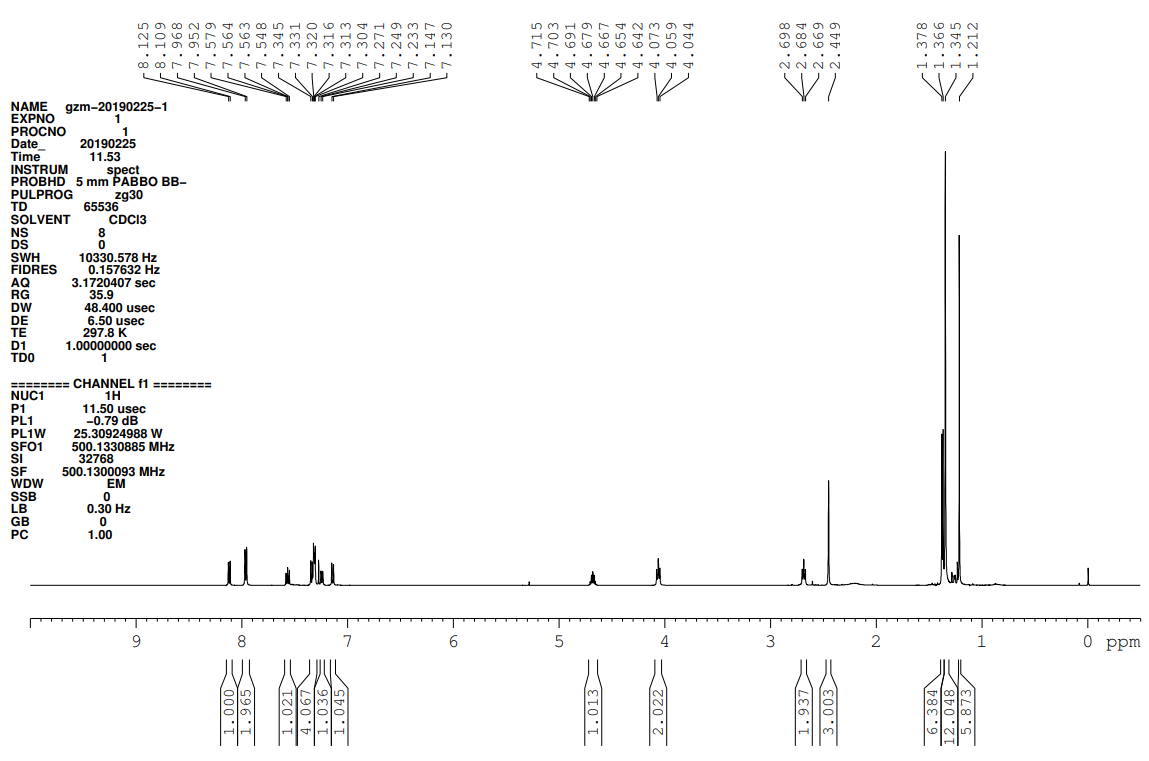


**Figure S1.** ^1^H-NMR spectrum of compound BPE **6**


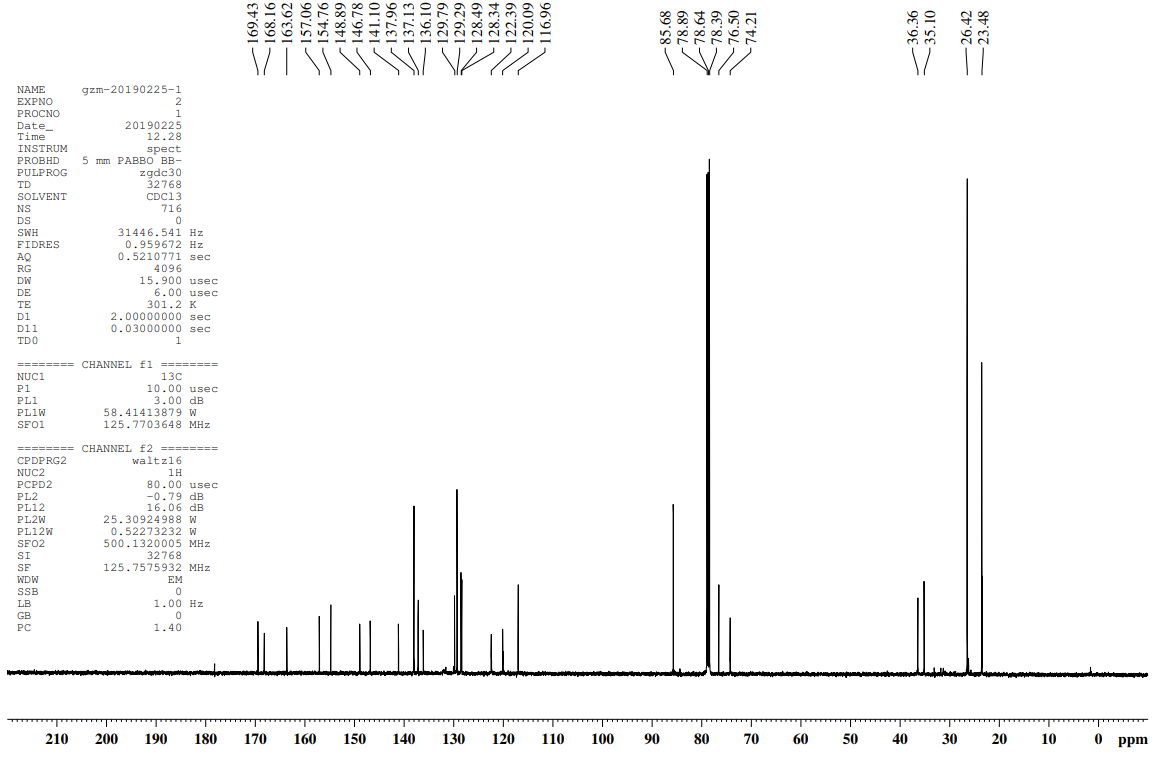


**Figure S2.** ^13^C-NMR spectrum of compound BPE **6**


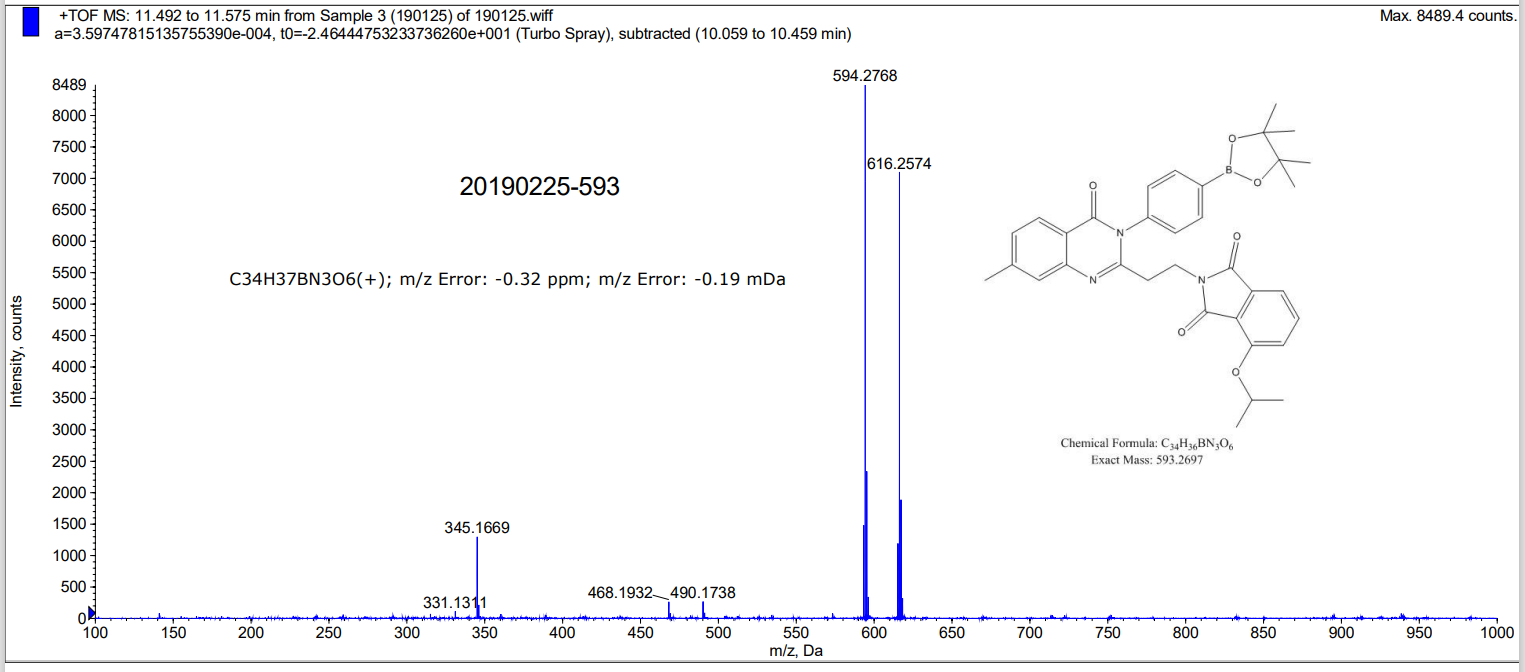


**Figure S3.** HRMS chromatograms of compound BPE **6**


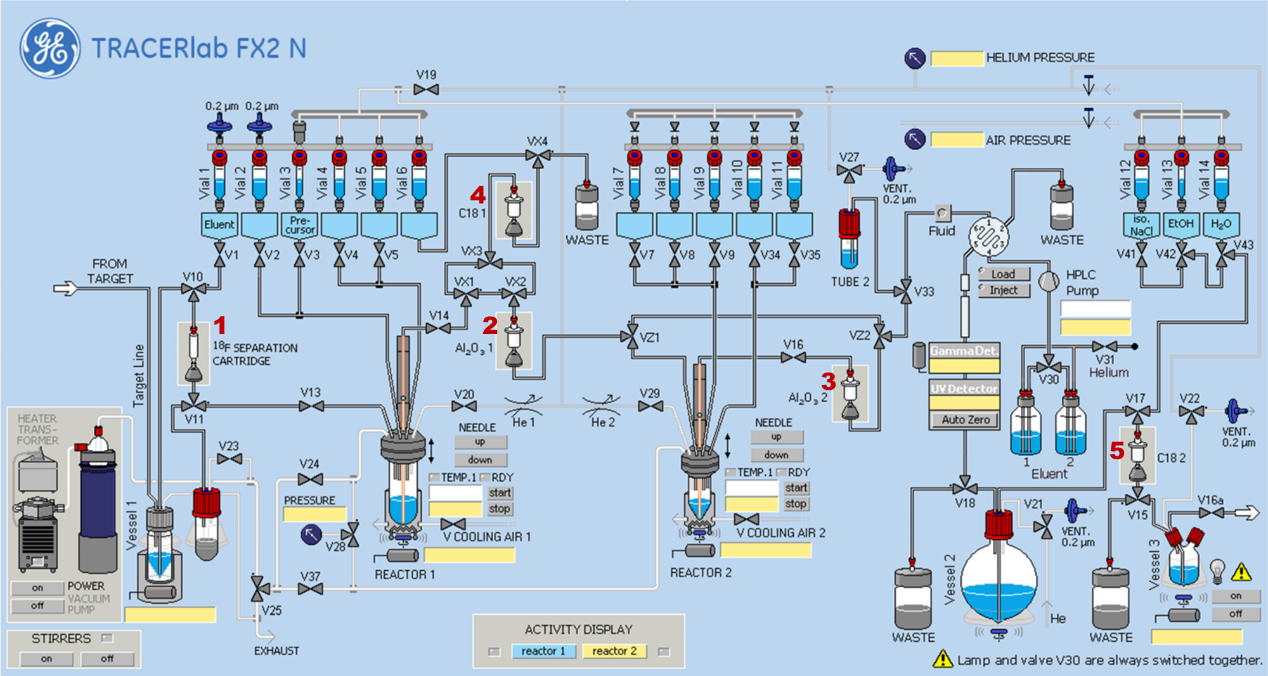


**Figure S4.** A schematic diagram of the GE TRACERlab FX2N radiosynthesis module

(A)


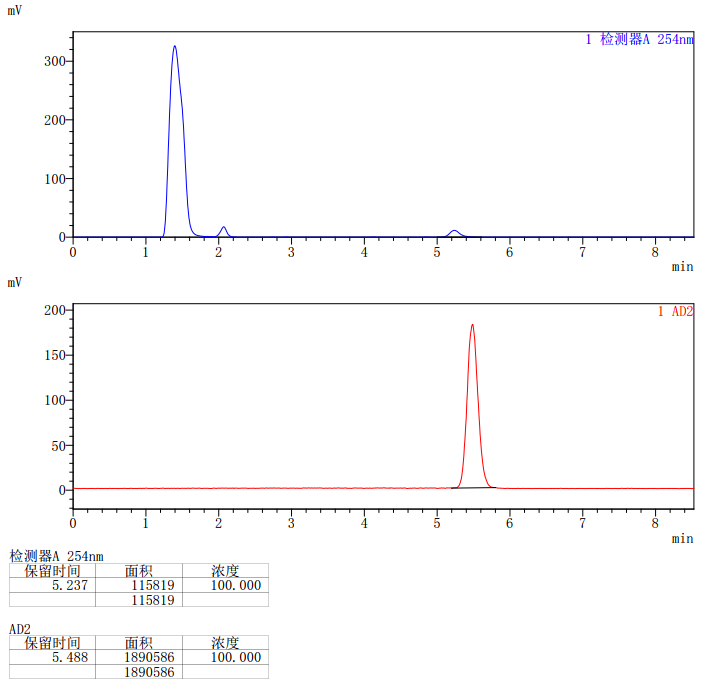


(B)


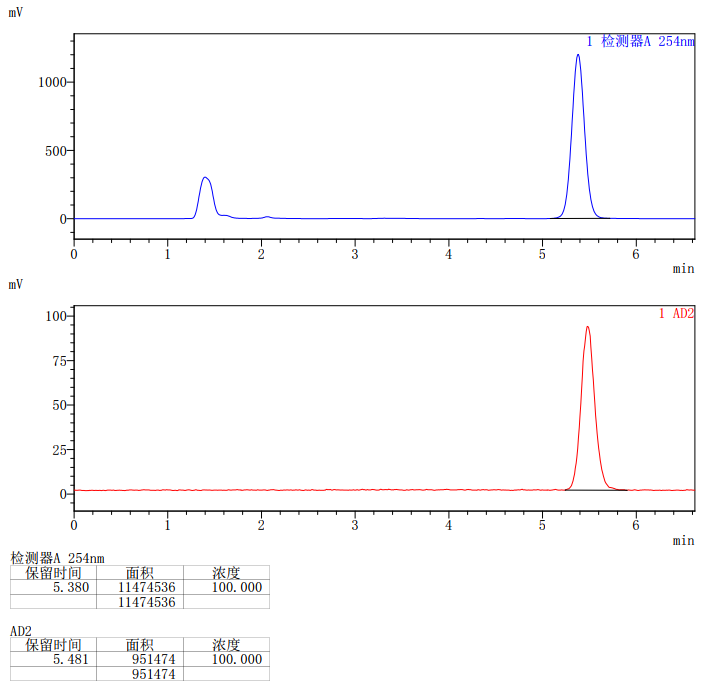


**Figure S5.** Chromatograms of [^18^F]P10A-1910 radiolabeled via *Method I*. (A) Injection of formulated [^18^F]P10A-1910; (B) co-injection with standard P10A-1910. Analytic column: GL Sciences, WondaSil C18-WR, 4.6×150 mm; mobile phase: MeCN/H_2_O = 70/30 (v/v); flow rate: 1.0 mL/min.

(A)


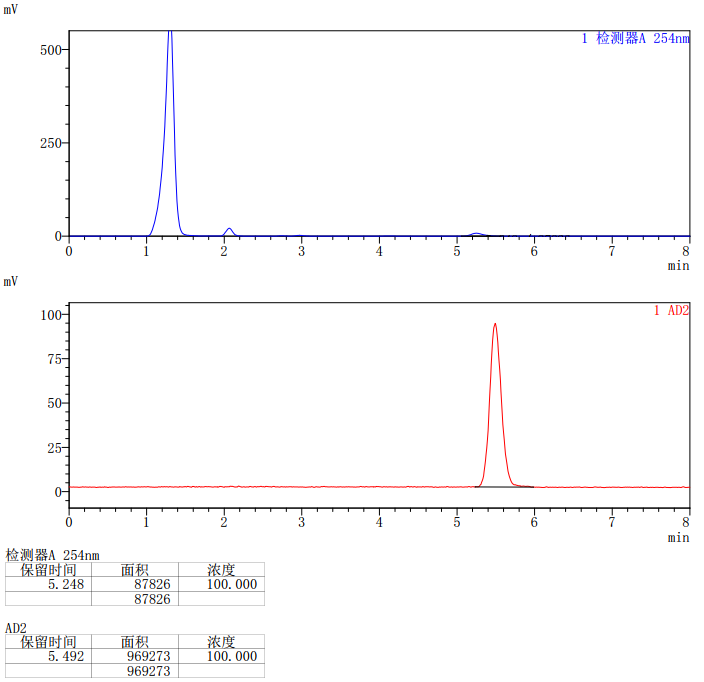


(B)


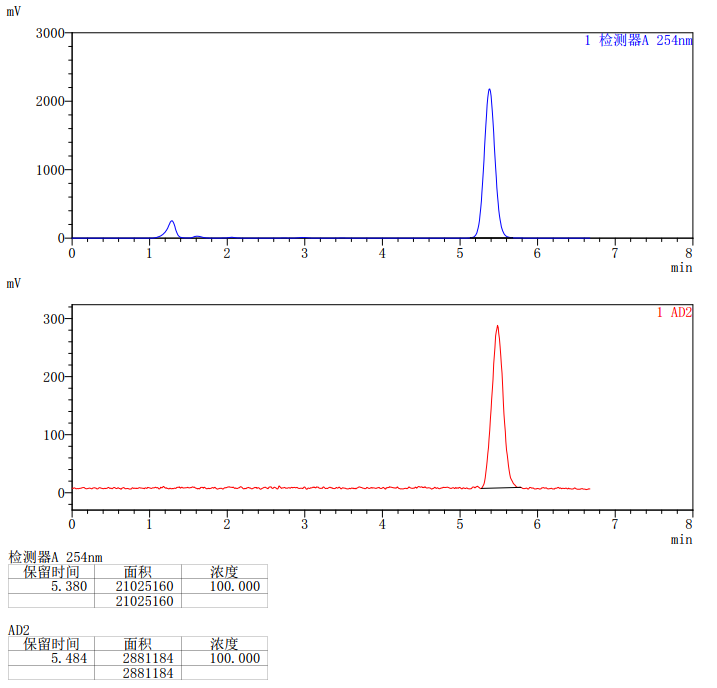


**Figure S6.** Chromatograms of [^18^F]P10A-1910 radiolabeled via *Method II*. (A) Injection of formulated [^18^F]P10A-1910; (B) co-injection with standard P10A-1910. Analytic column: GL Sciences, WondaSil C18-WR, 4.6×150 mm; mobile phase: MeCN/H_2_O = 70/30 (v/v); flow rate: 1.0 mL/min.

(i) Compound information

| **Compound ID** | **Molecular Weight** | **Mass (mg)** | **Mol (μmol)** | **Injected volume (mL)** |
| --- | --- | --- | --- | --- |
| P10A-1910 | 593.27 | 1.2 | 2.02268 | 0.02 |

(ii) Standard Curve

| **Serial No.** | **0** | **1** | **2** | **3** | **4** | **5** |
| --- | --- | --- | --- | --- | --- | --- |
| **Concentration (μmol/mL)** | 2.022688 | 0.202269 | 0.040454 | 0.020227 | 0.004045 | 0.002023 |
| **Mol (μmol)** | **0.040454** | **0.004045** | **0.000809** | **0.000405** | **0.000081** | **0.000040** |
| **UV Area (ave., *n* = 3)** | **30699249** | **3153972** | **648060** | **331393** | **116956** | **37353** |


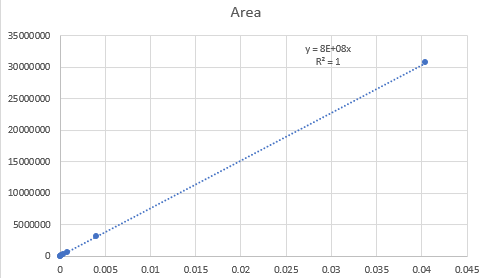


**(iii) Calculation of molar activity at the end of synthesis**

| **Type** | **Tracer Conc.**  **(mCi/mL)** | **Injected**  **volume (mL)** | **Injected**  **dose (Ci)** | **Slope** | **UV area** | **Cold mass**  **(μmol)** | **MA at EOS**  **(Ci/μmol)** |
| --- | --- | --- | --- | --- | --- | --- | --- |
| Method Ⅰ | 1.59 | 0.02 | 0.0000318 | 8×10^8^ | 12064 | 1.51×10^-5^ | **2.11** |
|  | 2.36 | 0.02 | 0.0000472 | 8×10^8^ | 20750 | 2.59×10^-5^ | **1.82** |
|  | 3.2 | 0.02 | 0.000064 | 8×10^8^ | 15730 | 1.97×10^-5^ | **3.25** |
| Method Ⅱ | 2.27 | 0.02 | 0.0000454 | 8×10^8^ | 15679 | 1.96×10^-5^ | **2.32** |
|  | 2.69 | 0.02 | 0.0000538 | 8×10^8^ | 20033 | 2.50×10^-5^ | **2.15** |
|  | 1.86 | 0.02 | 0.0000372 | 8×10^8^ | 14194 | 1.77×10^-5^ | **2.10** |

**Figure S7.** Calculation of molar activities of [^18^F]P10A-1910 at the end of formulation.


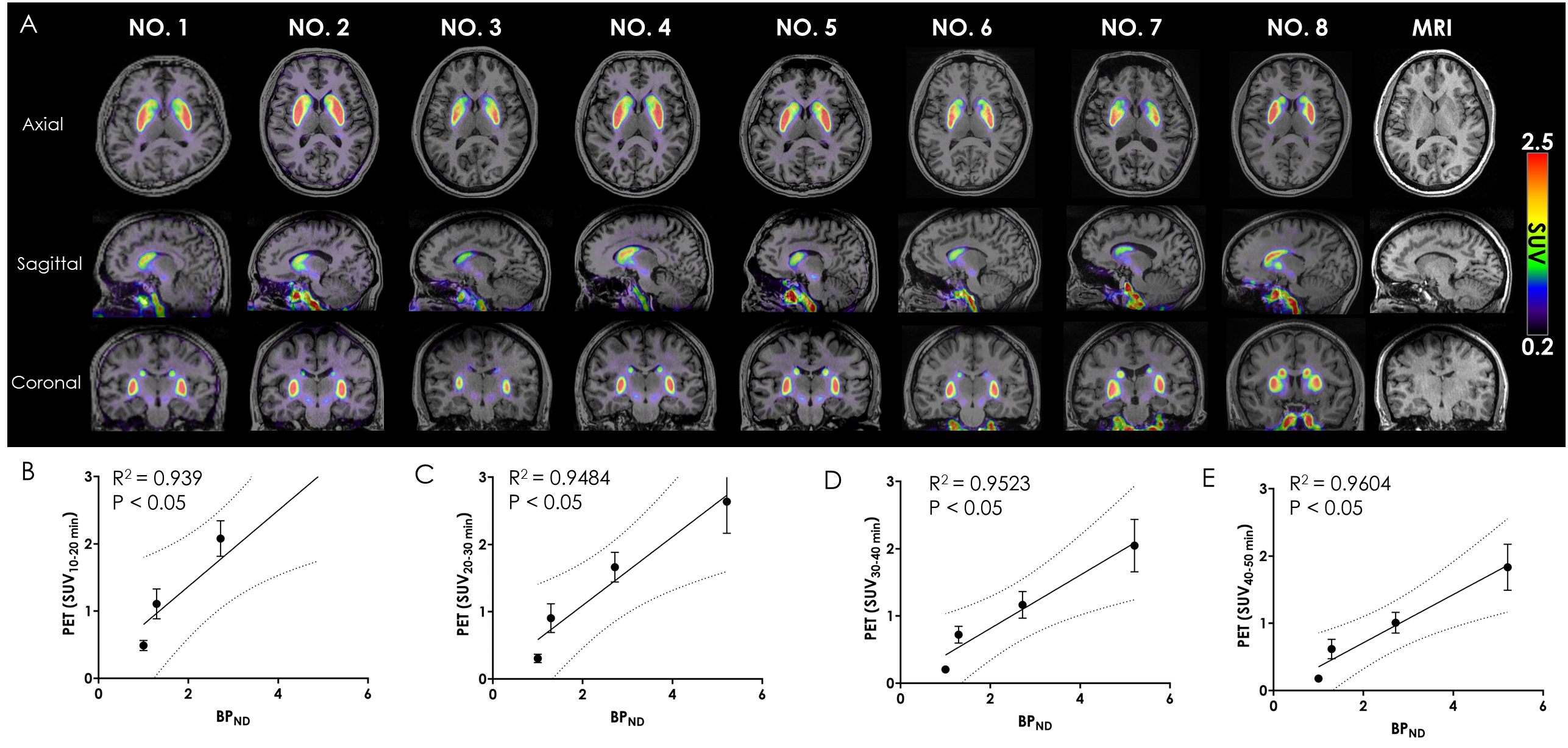


**Figure S8.** PET images in healthy volunteers and linear correlation analysis between SUV and *BP*_ND_. Data are presented as mean ± SD (*n* = 8). Strength and direction of associations were assessed by Spearman’s rank-order correlation (rs). *p* < 0.05 represents a statistical difference.
